# Cardiovascular Disease Prognostic Models in Latin America and the Caribbean: A Systematic Review

**DOI:** 10.1016/j.gheart.2019.03.001

**Published:** 2019-03

**Authors:** Rodrigo M. Carrillo-Larco, Carlos Altez-Fernandez, Niels Pacheco-Barrios, Claudia Bambs, Vilma Irazola, J. Jaime Miranda, Goodarz Danaei, Pablo Perel

**Affiliations:** ∗ Department of Epidemiology and Biostatistics, School of Public Health, Imperial College London, London, UK; † CRONICAS Centre of Excellence in Chronic Diseases, Universidad Peruana Cayetano Heredia, Lima, Peru; ‡ Facultad de Medicina “Alberto Hurtado”, Universidad Peruana Cayetano Heredia, Lima, Peru; § Department of Public Health and Advanced Center for Chronic Diseases (ACCDiS), Facultad de Medicina, Pontificia Universidad Católica de Chile, Santiago, Chile; ‖ Institute for Clinical Effectiveness and Health Policy (IECS), Buenos Aires, Argentina; ¶ Department of Global Health and Population, Harvard T.H. Chan School of Public Health, Boston, MA, USA; # Centre for Global Chronic Conditions, London School of Hygiene and Tropical Medicine, London, UK

## Abstract

**Background:**

Cardiovascular prognostic models guide treatment allocation and support clinical decisions. Whether there are valid models for Latin American and Caribbean (LAC) populations is unknown.

**Objective:**

This study sought to identify and critically appraise cardiovascular prognostic models developed, tested, or recalibrated in LAC populations.

**Methods:**

The systematic review followed the CHARMS (CHecklist for critical Appraisal and data extraction for systematic Reviews of prediction Modelling Studies) framework (PROSPERO [International Prospective Register of Systemic Reviews]: CRD42018096553). Reports were included if they followed a prospective design and presented a multivariable prognostic model; reports were excluded if they studied symptomatic individuals or patients. The following search engines were used: EMBASE, MEDLINE, Scopus, SciELO, and LILACS. Risk of bias assessment was conducted with PROBAST (Prediction model Risk Of Bias ASsessment Tool). No quantitative summary was conducted due to large heterogeneity.

**Results:**

From 2,506 search results, 8 studies (N = 130,482 participants) were included for qualitative synthesis. We could not identify any cardiovascular prognostic model developed for LAC populations; reviewed reports evaluated available models or conducted a recalibration analysis. Only 1 study included a Caribbean population (Puerto Rico); 3 studies were retrieved from Chile; 2 from Argentina, Brazil, Colombia, and Uruguay; and 1 from Mexico. Four studies included population-based samples, and the other 4 included people affiliated to a health facility (e.g., prevention clinics). Most studied participants were older than 50 years, and there were more women in 5 reports. The Framingham model was assessed 6 times, and the American College of Cardiology/American Heart Association pooled equation was assessed twice. Across the prognostic models assessed, calibration varied widely from one population to another, showing great overestimation particularly in some subgroups (e.g., highest risk). Discrimination (e.g., C-statistic) was acceptable for most models; for Framingham it ranged from 0.66 to 0.76. The American College of Cardiology/American Heart Association pooled equation showed the best discrimination (0.78). That there were few outcome events was the most important methodological limitation of the identified studies.

**Conclusions:**

No cardiovascular prognostic models have been developed in LAC, hampering key evidence to inform public health and clinical practice. Validation studies need to improve methodological issues.

## Highlights

• There has never been a cardiovascular prognostic model developed in LAC.
• Few studies have tested available models, but they have methodological limitations.
• Discrimination estimates were acceptable across studies.
• Calibration estimates showed important overestimation across studies.
• Many countries in Latin America do not have tools for cardiovascular prevention.

Cardiovascular diseases are the leading cause of death and disability, both globally and in Latin America and the Caribbean (LAC) 1, 2. These trends can be improved through different strategies, modifying the distribution of risk factors in the population as a whole, that is, a population-based prevention approach (e.g., sugar taxes), and through an overall risk-based prevention approach 3, 4. It has been argued that the latter is cost-effective and maximizes resources allocation (e.g., treatment) to those who most need them with minimal harm 5, 6. However, risk-based prevention needs accurate prognostic tools to identify the target population.

There is some evidence that available cardiovascular risk prediction equations do not perform well in LAC [7], where cardiovascular key risk factors such as diabetes seem to have different strength of association with cardiovascular events [8]. Therefore, it becomes necessary to identify which available equations have undergone local scrutiny and whether new local tools have been developed. Even though there have been efforts to summarize cardiovascular prognostic models 9, 10, they did not include studies written in Spanish or search engines with large LAC influence, hence reporting no results from LAC [10].

Therefore, whether available cardiovascular prognostic models have been tested or a new model has been derived in LAC, remains largely unknown. Consequently, we conducted a systematic review to summarize and critically appraise studies evaluating or generating prognostic models for cardiovascular outcomes conducted in LAC. In so doing, we provide a comprehensive list of available prognostic models, their strengths and limitations, as well as recommendations and identification of research gaps to be addressed to improve cardiovascular prevention in LAC.

## Methods

### Study design

The protocol for this systematic review of the literature was registered at PROSPERO (International Prospective Register of Systematic Reviews) (CRD42018096553) [11]. This work adheres to the PRISMA (Preferred Reporting Items for Systematic Reviews and Meta-Analyses) guidelines (see the PRISMA Checklist in the Online Appendix) and CHARMS (CHecklist for critical Appraisal and data extraction for systematic Reviews of prediction Modelling Studies) framework 12, 13, 14. Following the CHARMS framework, we sought prognostic models that predict the risk of having a cardiovascular (nonfatal and fatal) outcome in a pre-defined period of time to be used in the general population to guide prevention or treatment recommendations (Table 1). We exclusively focused on reports that included LAC populations.

**Table 1:** Review framework according to the CHARMS checklist

Item	Criterion
Prognostic or diagnostic	Prognostic, i.e., future events.
Scope	Prognostic models to inform clinicians (and general population) about the risk of a person to develop a nonfatal/fatal cardiovascular event in a pre-defined period.
Type of prediction models	Prognostic models with and/or without external validation.
Prediction target population	General population, men and women.
Outcome of interest	Any nonfatal or fatal cardiovascular event, including myocardial infarction, stroke, or cardiovascular death; these outcomes could have been studied independently or as a composite endpoint.
Prediction period	Any (e.g., 10 yrs).
Intended moment to apply the prediction tool	Prognostic tool to be used in primary prevention to assess cardiovascular risk and thus guide prevention/treatment.

CHARMS, CHecklist for critical Appraisal and data extraction for systematic Reviews of prediction Modelling Studies.

### Eligibility criteria

Inclusion criteria comprised the following: 1) The overall study design had to be prospective; this included studies that turned into prospective cohorts, even though they were not originally designed as such. For example, a cross-sectional survey/study in which participants were looked up in death/hospital registries after some years. 2) The study reported a multivariable (i.e., at least 2 variables or predictors) model to predict the risk of developing a nonfatal and/or fatal cardiovascular event (e.g., stroke, myocardial infarction, and death) in an individual of the general population. On the other hand, exclusion criteria included the following: 1) the study population targeted symptomatic (e.g., emergency care) or only patients with specific pathologies (e.g., chronic kidney disease); and 2) the study population targeted people who, by the time of the baseline assessment, had already experienced a cardiovascular outcome (e.g., stroke).

### Information sources

On July 15, 2018, a systematic search of publications was conducted using 5 search engines: Ovid (EMBASE and MEDLINE), Scopus, SciELO and LILACS; the latter 2 are LAC-specific. The search terms were based on a recent systematic review, which only included EMBASE, MEDLINE, and articles in English [10]. In addition, terms regarding LAC countries were included (i.e., country names). No additional filters (e.g., language or publication year) were set. The list of search terms is presented in the Online Appendix.

### Search

Before titles and abstract screening, 2 reviewers (RMC-L and NP-B) agreed on a standard approach. Two random samples of 50 search results were selected for training purposes. Reviewers screened these titles and abstracts and the inter-rater agreement and kappa estimator were computed, aiming for an inter-rater agreement of at least 90% (see “Training of reviewers” in the Online Appendix). After this standardization process, the 2 reviewers (RMC-L and NP-B) screened all titles and abstracts following the pre-specified framework and selection criteria. Discrepancies were solved by a third reviewer independently (CA-F). After the title and abstract selection, full text of selected reports was sought and analyzed by 2 reviewers (RMC-L and CA-F) following the same above-mentioned selection criteria; discrepancies were solved by consensus between these reviewers. These selection processes—titles and abstracts as well as full texts—were conducted using the online tool Rayyan-a [15]. With the final list of studies to be included for qualitative synthesis, 2 reviewers (RMC-L and CA-F) extracted relevant information in a pre-specified form developed by the authors based on the CHARMS framework 13, 14. Meta-analysis was not conducted because of the large heterogeneity among studies. Results were summarized qualitatively and relevant point estimates (e.g., C-statistics) are presented.

### Risk of bias

The PROBAST tool for risk of bias appraisal was used 16, 17, 18. This tool was applied by 1 reviewer (RMC-L). The PROBAST (Prediction model Risk Of Bias ASsessment Tool) tool has been designed to assess risk of bias of multivariate prognostic models in 4 domains: 1) participants; 2) predictors; 3) outcome; and 4) analysis. The criteria within each domain have 5 possible answers: yes; probably yes; probably no; no; and no information. A positive answer suggests no risk of bias. The domains had 3 potential outcomes: low; high; or unclear risk of bias.

## Results

### Study selection

The search retrieved 2,506 results. After removing duplicates, 2,420 titles and abstracts were screened, and of these 2,403 were excluded. Of the 17 reports studied in detail, 8 reports (N = 130,482 participants) were selected for qualitative synthesis (see the “Extraction form” in the Online Appendix). There was the same number of reports and studies.

### Study characteristics

**Figure 1:**
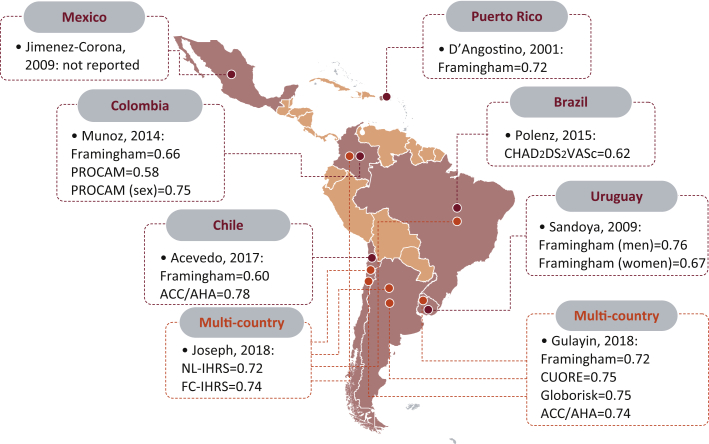
**Discrimination estimates for each prognostic model by country.** Confidence intervals, when reported, are presented in Online Table 1. In Acevedo [19], the outcome was cardiovascular mortality (did not include nonfatal events). **Brown dots** represent studies conducted with populations in 1 country alone, and **orange dots** are for multicountry studies. PROCAM is a prognostic model for men, the “PROCAM (sex)” indicates the adjusted model so that it can be used for men and women. Figure template from http://yourfreetemplates.com (see “Details for preparation of Figure 1” in the Online Appendix). ACC/AHA, American College of Cardiology/American Heart Association; CHA_2_DS_2_VASc, Congestive Heart Failure, Hypertension, Age ≥75 Years, Diabetes Mellitus, Prior Stroke or Transient Ischemic Attack or Thromboembolism, Vascular Disease, Age 65 to 74 Years, Sex; CUORE, Continuous Ultrafiltration for Congestive Heart Failure; FC-IHRS, fasting cholesterol INTERHEART risk score; INTERHEART, Effect of Potentially Modifiable Risk Factors Associated With Myocardial Infarction in 52 Countries; NL-IHRS, nonlaboratory INTERHEART risk score; PROCAM, Prospective Cardiovascular Münster.

Five of the 8 reports were published in Latin-American journals 19, 20, 21, 22, 23, with 3 of them written in Spanish 19, 22, 23. The oldest report was published in 2001 [24] and the newest ones in 2018 20, 25. Six reports were conducted in 1 country only 19, 21, 22, 23, 24, 26, and there were 2 multicountry efforts including Argentina, Chile, and Uruguay [20], as well as Argentina, Brazil, Chile, and Colombia (Fig. 1) [25]. The country that has been mostly included in this review was Chile (3 of 8) 19, 20, 25; Argentina, Brazil, Colombia, and Uruguay were studied in 2 reports 20, 22, 23, 25, 26; Puerto Rico [24] and Mexico [21] were included in 1 report (Fig. 1). None of the reviewed studies reported adherence to the TRIPOD (Transparent Reporting of a Multivariable Prognostic Model for Individual Prognosis or Diagnosis) statement.

Four studies collected baseline information in the 2000s 19, 20, 25, 26, 3 in the 1990s 21, 22, 23, and 1 between 1965 and 1968 [24]. One-half of the selected studies included population-based samples 20, 21, 24, 25, whereas the other one-half included people that were somehow affiliated with health care facilities 19, 22, 23, 26. For example, Muñoz et al. [22] included individuals who attended a primary prevention clinic at the Central Military Hospital in Colombia. Likewise, Acevedo et al. [19] enrolled individuals who voluntarily attended a cardiology prevention program in Chile. Women accounted for more than one-half of the study population in 5 reports 20, 21, 22, 23, 25; in 1 report the women-to-men ratio was 1 [26]; and in 2 reports, men accounted for a larger share of the study population 19, 24. Although it was not possible to extract baseline age information from 1 study [20], the others had either a mean of, or a larger proportion of, people ≥50 years old 19, 22, 23, 24, 25, 26; in the report by Jimenez-Corona et al. [21], the mean age was 47 years.

Across the studies summarized herein, the mean sample size was 16,310 (SD: 37,107.4). Sample size was over 1,000 people in 7 of the studied reports, and 1 report included 468 individuals [26]. The largest sample sizes were from multicountry endeavors 20, 25. The only study conducted in the Caribbean in the mid-1960s also had a large sample size (n = 8,713) [24]. One study did not report follow-up time duration [25], and another reported 10 years of follow-up or censoring/event [22]; in general, follow-up time ranged from 12 ± 4 months [26] to 9.2 ± 2.1 years [23].

Most reports included the same outcomes as those of the original prognostic model being studied (e.g., Framingham model); however, although the original model included both nonfatal and fatal cardiovascular events, 1 report could only analyze the latter [19]. All reports included the same predictors of the original model, and these were assessed following standard procedures such as clinical examination or laboratory tests. Further details about the characteristics of the summarized reports, including methods for predictors and outcomes ascertainment, are shown in Table 2 and the Extraction Form in the Online Appendix.

**Table 2:** Methodological characteristics of the reviewed prognostic models

Study, Year (Ref. #)	Predictors Ascertainment	Outcome Details	Outcome Ascertainment	Mean Follow-Up (yrs)∗	Baseline Sample	Outcome Events	Original Prediction Model Being Tested
Polenz, 2015 [26]	Two physicians independently performed clinical assessment and reviewed electronic medical records.	All-cause mortality, stroke, transient ischemic attack, acute myocardial infarction, and new atrial fibrillation/flutter.	A specialist validated each outcome (e.g., stroke by a neurologist).	1.3 (12 ± 4 months)	468	15	CHA_2_DS_2_VASc
Muñoz, 2014 [22]	A researcher extracted all the information from health records.	Total coronary disease: coronary death; myocardial infarction; angina pectoris, coronary insufficiency. Hard coronary disease: coronary death and myocardial infarction.	By a researcher who did not have access to baseline information. Health records of people suspected to have had a coronary event were verified by an internal medicine physician, who defined whether these were either total or hard coronary diseases. Cause of death was based on death certificates or discharge records.	10 yrs, event or censoring	1,013	61	Framingham, PROCAM
Gulayin, 2018 [20]	Blood pressure, measured after a 5-min rest using a mercury or aneroid sphygmomanometer; average of 3 readings was used. Blood samples were withdrawn after ≥10 h of fasting.	Angina pectoris, nonfatal/fatal myocardial infarction, nonfatal/fatal stroke, coronary artery, carotid or peripheral revascularization, heart failure, and sudden death.	Events were confirmed by an internal medicine or cardiology specialist after verification of the event-specific record.	Median = 2.2 (IQR: 1.9–2.8)	6,364	60	CUORE, Framingham, Globorisk, ACC/AHA Pooled Equation
Sandoya, 2009 [23]	Interviews for smoking and medication use. Blood pressure measured with a semi-automatic validated instrument during a resting period, using the mean of 3 measurements separated at least 2 min. Blood samples were withdrawn after a 12-h fasting period and analyzed in a central laboratory.	Ischemic disease as ICD-10—I21, I20, I20.9, I46—or revascularization.	Based on discharge diagnosis on medical records, when needed telephone communications were held with the participants or relatives.	9.2 ± 2.1	1,110	72	Framingham
Acevedo, 2017 [19]	Blood samples withdrawn after a 12-h fasting period from venous samples. All participants were interviewed. Blood pressure was measured according to JNC VII.	Nonfatal/fatal myocardial infarction, nonfatal/fatal stroke, or other cardiovascular event.	Deaths registries were obtained. No information on ascertainment of nonfatal events; it is reported that the analyses were based on mortality as the outcome (cardiovascular mortality).	7 ± 3	3,284	34	ACC/AHA Pooled Equation; Framingham and Framingham Chileno.
Jiménez-Corona, 2009 [21]	Standard questionnaires were used. Blood pressure was measured 3 times after a 5-min rest using a random 0 sphygmomanometer; the mean of the last 2 records was used. Fasting serum total cholesterol and HDL were determined by cholesterol-esterase.	Nonfatal/fatal myocardial infarction.	By resting ECG or by death certificate. ECG were interpreted according to the Minnesota code, including possible and probable myocardial infarctions. Death certificates in which the underlying cause of death was ICD-10 410–410.9.	Median = 6.2 (range 0.2–9.8)	1,667	58	Framingham by Wilson et al., and by Anderson et al.
Joseph, 2018 [25]	No details provided.	Cardiovascular death, myocardial infarction, stroke, heart failure, or revascularization (percutaneous coronary intervention or coronary artery bypass).	Participants or relatives were interviewed for cardiovascular events. All events were reviewed at each study site using supporting documentation, verbal autopsies, or medical records; standard definitions were used.	4.89 (2.24)	100,475 (NL-IHRS) 107,863 (FC-IHRS)	352	NL-IHRS and FC-IHRS
D'Agostino, 2001 [24]	No details provided.	Coronary death or myocardial infarction.	No details provided.	No details provided.	8,713	No details provided.	Framingham

ACC/AHA, American College of Cardiology/American Heart Association; CHA_2_DS_2_VASc, Congestive Heart Failure, Hypertension, Age ≥75 Years, Diabetes Mellitus, Prior Stroke or Transient Ischemic Attack or Thromboembolism, Vascular Disease, Age 65 to 74 Years, Sex; CUORE, Continuous Ultrafiltration for Congestive Heart Failure; ECG, electrocardiography; FC-IHRS, fasting cholesterol INTERHEART risk score; HDL, high-density lipoprotein; ICD-10, *International Classification of Diseases*, 10th revision; INTERHEART, Effect of Potentially Modifiable Risk Factors Associated With Myocardial Infarction in 52 Countries; IQR, interquartile range; JNC VII, Seventh Report of the Joint National Committee on Prevention, Detection, Evaluation, and Treatment of High Blood Pressure; NL-IHRS, nonlaboratory INTERHEART risk score; PROCAM, Prospective Cardiovascular Münster.

∗ Unless otherwise indicated.

### Cardiovascular prognostic models

#### Characteristics

None of the reviewed reports developed a prognostic model for cardiovascular events based on LAC populations; conversely, reviewed reports tested available prognostic models 19, 21, 22, 23, 26 and some of them pursued recalibration strategies 20, 24, 25.

The most frequently studied prognostic model was the Framingham risk prediction equation (6 times) 19, 20, 21, 22, 23, 24, followed by the American College of Cardiology/American Heart Association Pooled Cohorts Equation (2 times) 19, 20; all other prognostic models were studied once: the nonlaboratory INTERHEART (Effect of Potentially Modifiable Risk Factors Associated With Myocardial Infarction in 52 Countries) risk score and the fasting cholesterol INTERHEART risk score [25]; Globorisk [20]; CUORE (Continuous Ultrafiltration for Congestive Heart Failure) [20]; PROCAM (Prospective Cardiovascular Münster) [22]; and CHA_2_DS_2_VASc (Congestive Heart Failure, Hypertension, Age ≥75 Years, Diabetes Mellitus, Prior Stroke or Transient Ischemic Attack or Thromboembolism, Vascular Disease, Age 65 to 74 Years, Sex) [26].

Most reports studied prognostic models with few outcome events per predictor. One study had 352 outcome events [25], and in 6 reports this figure ranged from 15 [26] to 72 [23] (Table 2). In addition, none reported a formal sample size estimation or whether the number of outcome events was adequate given the number of predictors.

Missing data was handled by conducting a complete-case analysis in 5 reports 20, 21, 23, 25, 26, only 1 conducted multiple imputation [22], and 2 did not provide information about this matter 19, 24. Only 1 of the reviewed reports conducted bootstrap analysis to compute the confidence intervals of the area under the receiver operator curve [19].

#### Performance

Most reports provided estimates of calibration and discrimination 20, 22, 23, 24, 25; 2 also reported classification metrics such as positive/negative likelihood ratio 20, 26. Calibration and discrimination estimates are presented in Table 3 and Figure 1 (Online Table 1). Although all discrimination estimates (e.g., C-statistics) were reported with wide confidence intervals, the highest discrimination metric achieved was for the American College of Cardiology/American Heart Association (ACC/AHA) pooled risk equation by Acevedo et al. [19]; however, they did not include all the outcomes of the original model so that this estimate should be interpreted cautiously.

**Table 3:** Prediction properties of the prognostic models as reported in the reviewed reports

Study, Year Ref. #	Calibration	Discrimination	Classification Measures
Polenz, 2015 [26]	No details provided.	C-statistic = 0.62 (95% CI: 0.58–0.67)	At a score of ≥6 points + LR = 3.45, −LR = 0.78, sensitivity = 28.6, specificity = 91.7 for the occurrence of stroke or TIA; +LR = 3.35, −LR = 0.79, sensitivity = 27.3, specificity = 91.9 for stroke, TIA, and death.
Muñoz, 2014 [22]	Framingham: overestimation; for people at low and intermediate risk, the relationship between expected and observed was 1.31; for people at high risk, the absolute difference between the proportion of expected and observed events was 17.4. PROCAM: similar findings for all risk groups; for people at low and intermediate risk the absolute difference between percentages of expected and observed events was <3%; poor calibration (overestimation) for people at high risk. PROCAM adjusted for sex (so that can be used in men and women) showed similar calibration properties.	Framingham: AUC = 0.6584 (95% CI: 0.6258–0.6907). PROCAM: AUC = 0.5819 (95% CI: 0.5238–0.6385). PROCAM adjusted for sex: AUC = 0.7446 (95% CI: 0.7142–0.7740).	No details provided.
Gulayin, 2018 [20]	They reported the β slope for calibration. CUORE: *y* = 1.012*x* − 0.0036. Framingham: *y* = 1.0956*x* − 0.014. Globorisk: *y* = 1.3718*x* − 0.0066. ACC/AHA pooled equation: *y* = 0.5103*x* + 0.0095.	CUORE: C-statistic = 0.751 and Harrell's C index = 0.752. Framingham: C-statistic = 0.719 and Harrell's C index = 0.722. Globorisk: C-statistic = 0.753 and Harrell's C index = 0.736. ACC/AHA pooled equation: C-statistic = 0.736 and Harrell's C index = 0.743.	CUORE: sensitivity = 73% and specificity = 69%. Framingham: sensitivity = 81% and specificity = 51%. Globorisk: sensitivity = 75% and specificity = 60%. ACC/AHA pooled equation: sensitivity = 75% and specificity = 58%.
Sandoya, 2009 [23]	Hosmer-Lemeshow for men was 6.82 (*p* = 0.56) and for women was 5.09 (*p* = 0.64).	AUC for men was 0.76 (95% CI: 0.69–0.82) and for women was 0.67 (95% CI: 0.56–0.78).	No details provided.
Acevedo, 2017 [19]	No details provided.	ACC/AHA pooled equation: AUC = 0.78 (95% CI: 0.68–0.84). Framingham: AUC = 0.60 (95% CI: 0.52–0.74). Framingham Chileno: AUC = 0.67 (95% CI: 0.60–0.79).	No details provided.
Jiménez-Corona, 2009 [21]	The ratio of predicted/observed rates using the first equation (Framingham by Wilson et al.) was 1.84 (95% CI: 1.15–2.53) in men and 1.55 (95% CI: 1.01–2.08) in women; the ratio using the second equation (Framingham by Anderson et al. [27]) was 3.17 (95% CI: 1.67–4.68) in men and 1.57 (95% CI: 1.67–2.17) in women.	No details provided.	No details provided.
Joseph, 2018 [25]	Original NL-IHRS: slope = 0.87 (0.77–0.98), intercept = −4.43 (−4.75 to 4.29); for the recalibrated version these parameters were 1 (0.87–1.13) and 0 (−0.48 to 0.48). Original FC-IHRS: slope = 1.11 (0.97–1.24), intercept = −4.35 (−4.49 to 4.21); for the recalibrated version these parameters were 1 (0.88–1.12) and 0 (−0.45 to 0.45).	Original NL-IHRS: C-statistic = 0.72 (0.69–0.75); and so was for the recalibrated version. Original FC-IHRS: C-statistic = 0.74 (0.71–0.77) and so was for the recalibrated version.	No details provided.
D'Agostino, 2001 [24]	Best chi-square using the Puerto Rico study's means on the risk factors and the Puerto Rico study's CHD incidence = 7.2.	In Hispanic population, the best Cox (applying the Cox model developed on the Puerto Rico study's data): C-statistic = 0.72.	No details provided.

AUC, area under the curve; CHD, coronary heart disease; CI, confidence interval; LR, likelihood ratio; TIA, transient ischemic attack; other abbreviations as in Table 2.

### Risk of bias

In the participants, predictors, and outcome domains of the risk of bias, all the reports were deemed to be of low risk; the outcomes were clearly defined and agreed with those of the original model except for 1 study that addressed the American College of Cardiology/American Heart Association Pooled Cohorts Equation [19]. The fourth criteria—analysis—was troublesome for the reviewed reports largely because the few number of outcome events, yielding a limited outcome-predictors ratio, and for conducting complete-case analysis rather than multiple imputation process. A summary of the risk of bias analysis is shown in Table 4, and details on each criterion across domains are provided in Risk of Bias (PROBAST) in the Online Appendix.

**Table 4:** Risk of bias assessment

Study, Year	Risk of Bias RoB				Applicability			Overall	
	Participants	Predictors	Outcome	Analysis	Participants	Predictors	Outcome	Risk of Bias	Applicability
Polenz, 2015 [26]	Low	Low	Low	High	High	Low	Low	High	High
Muñoz, 2014 [22]	Low	Low	Low	High	High	Low	Low	High	High
Gulayin, 2018 [20]	Low	Low	Low	High	Low	Low	Low	High	Low
Sandoya, 2009 [23]	Low	Low	Low	High	High	Low	Low	High	High
Acevedo, 2017 [19]	Low	Low	High	High	High	Low	Low	High	High
Jiménez-Corona, 2009 [21]	Low	Low	Low	High	Low	Low	Low	High	Low
Joseph, 2018 [25]	Low	Low	Low	High	Low	Low	Low	High	Low
D'Agostino, 2001 [24]	Low	Low	Low	High	Low	Low	Low	High	Low

PROBAST, Prediction model Risk Of Bias ASsessment Tool.

In the risk of bias assessment, low means low risk of bias, high means high risk of bias, and unclear when it was not possible to assess the risk of bias. In the applicability section, high means high concern for applicability, low means low concern for applicability, and uncertain when it was not possible to assess the applicability. Risk of bias conducted with the PROBAST tool 16, 17, 18.

## Discussion

### Summary of evidence

Although cardiovascular prognostic models have been summarized by global systematic reviews, none of them found models or efforts undertaken in, and for, LAC populations 9, 10. This work complements these reviews with evidence from LAC and clearly demarcates that there is scope for the improvement of cardiovascular risk prediction in the LAC region. Eight studies were selected for qualitative synthesis: none developed a prognostic model, only 1 was conducted in the Caribbean [24], and many have major limitations with regards to sample size and analysis, for example, limited number of outcome events. The Framingham and the American College of Cardiology/American Heart Association Pooled Cohorts Equation were the most studied tools. Calibration estimates changed substantially from one population to another, with serious overestimation in some cases, that is, individuals categorized as high risk when they were not. Discrimination was acceptable in many reports, particularly for the American College of Cardiology/American Heart Association Pooled Cohorts Equation. Based on these findings, and the heterogeneous health profile of LAC populations, it is premature to strongly advocate for 1 prognostic model in LAC.

### Limitations at study level

Most studies analyzed few outcome events, which could account for wide confidence intervals, but most importantly for lack of power to make strong conclusions. Just recently, new approaches for estimating sample size or adequate number of events per predictors in the prognostic models have been proposed 28, 29. Future cardiovascular prognostic work could formally test these requirements. Joseph et al. [25] conducted the work with the “largest” outcome events per predictor ratio (ratio = 16). Nonetheless, and even though they had a model without laboratory variables, the model had over 20 predictors, which would make it troublesome to use in the field because it will require much information. In addition, some of the predictors were about diet profile [25], which could require further knowledge about local foods, hence making these questions difficult to ascertain.

It seemed that the reviewed studies made a great effort to conduct follow-up rounds and to accurately capture the outcomes of interest; however, 1 study could not adjudicate nonfatal outcomes even though the model being evaluated needed them [19]. This highlights the necessity for national health registries, at least of major events of noncommunicable diseases. These could inform health authorities, as well as researchers who will ultimately provide evidence to advise public health and clinical practice.

### Strengths and limitations at the review level

The research question and search strategy were defined following international guidelines for systematic reviews of prognostic models 13, 14. The search terms were based on previous systematic reviews and followed recommendations for finding prognostic studies 9, 10, 30. In addition, risk of bias was formally assessed with a validated tool 16, 17, 18. Nevertheless, this work is subject of several limitations. First, given the results about heterogeneity and the low number of reviewed reports, a quantitative summary (e.g., meta-analysis) was not be conducted. Second, we could have further reviewed gray reports, such as graduate programs dissertations of LAC universities. Although this could have retrieved more results, we doubt these would have been of greater quality than the published works herein analyzed. Therefore, we would have still not reached a strong recommendation for (or against) a given prognostic model.

### Other relevant publications

To summarize the strongest evidence on prognostic research we focused on prospective studies. Nonetheless, it seemed fair to also acknowledge other endeavors in LAC that, despite following different study designs, have still provided relevant evidence.

For example, Icaza et al. [31] adapted the Framingham equation using population-based estimates on risk factors and incidence of cardiovascular events based on national registries in Chile. This work has informed clinical and research practice in Chile. More recently, a population-based cohort was initiated in Maule, Central Chile. The MAUCO (Maule Cohort) study aims to enroll 10,000 people from 3 to 74 years old who will be followed for at least 10 years, with outcome measurements focused on cardiovascular diseases and cancer. The prospective design of MAUCO offers a unique opportunity to develop local prognostic models for the Chilean population [32]. Similar efforts in Peru and Argentina may provide solid evidence for these countries 33, 34, though a regional approach will still be missing.

The systematic search yielded 1 additional work from Central America. This was a bold project that attempted to generate a new prognostic model for myocardial infarction using case-control data from Costa Rica [35]. Despite the design limitations, they included a key item among their predictors: socioeconomic status. This relevant health determinant has been systematically excluded from many prognostic models, though just recently it has been highlighted by a novel model developed for New Zealand [36]. LAC is a region with large socioeconomic inequalities, and their effect on cardiovascular outcomes may be important. Therefore, should a new cardiovascular prognostic model be developed for LAC, a marker of socioeconomic status should be at least tested among other potential predictors.

Although gray reports were beyond the scope of our search, a master's dissertation was also retrieved by our search strategy; the outcome was coronary artery disease and included 349 individuals in Brazil [37]. This work included genetic predictors [37], which although relevant and research on this field should be fostered, it is perhaps premature to have prognostic models with predictors that are not widely available.

Finally, the Globorisk investigators developed cardiovascular risk charts for 182 countries including those in LAC 38, 39. Even though this was an example of global collaboration and comprehensive research methods, the risk charts were developed using risk prediction associations (e.g., hazard ratios) from cohorts mostly in the United States. This may limit the applicability of this model to other world regions including LAC, calling for updates to this and other models with weights (i.e., hazard estimates) retrieved from LAC studies.

### Research gaps

This review identified a lack of cardiovascular prognostic models developed in and for LAC populations, which needs urgent attention. Research funders, along with regional and local health agencies, should support new or ongoing prospective cohort studies to address this gap. This does not necessarily mean establishing a new multicountry or region-wide cohort but supporting further follow-up rounds of existing cohorts, particularly of population-based cohorts. In addition, regional and local health authorities could work to facilitate access to national health/death registries for research purposes. This way, large cross-sectional epidemiological studies and surveys could access data on cardiovascular nonfatal and fatal outcomes, in other words, entering the world of health big data in LAC. In the meantime, cohort data pooling may be a feasible solution.

Another relevant research gap observed by this review is the dearth of studies from the Caribbean and Central America regions. Besides a case-control study (Costa Rica) [35] and a prospective cohort, the baseline assessment of which was in the 1960s (Puerto Rico) [24], no additional results were retrieved for these regions. This calls for investigators to strengthen local research capacity and to generate, reuse, or update available cohorts in the Caribbean and Central America.

In terms of methods, this review found that 1 study followed multiple imputation to account for missing data; also, there were a low number of outcome events and some predictors were categorized when they could have been used as continuous variables 40, 41. These methodological approaches could hide a lack of training or expertise in validation (and development) of prognostic models. Despite abundant methodological evidence (e.g., statistical primers [42] and reviews 43, 44, 45, 46) on prognosis models, these are mostly in English, which could be a limitation for some researchers in LAC. In terms of capacity building, existing training programs in epidemiology and related fields should include prognostic research methods.

Reporting was also heterogeneous across studies. It would be impossible for studies published before 2015 to have adhered to the TRIPOD statement [47], but neither did the post-2015 papers. This again calls for investigators to improve prediction research capacity in LAC.

### Implications for public health

Where poor-quality health care could be worse than any health care [48], the health system should secure adequate interventions to prevent diseases and complications. This ranges from highly complex treatments to prevention strategies. Among the latter, estimating the absolute risk of an event might guide the allocation of treatment and preventive measures in favor of high-risk individuals. However, if prediction tools are not accurate enough, people who do not need treatment would unnecessarily receive medication and people who need it would not be identified. Considering this systematic review, local and regional health authorities, researchers, and practitioners should understand the pitfalls of using available cardiovascular prognostic models in LAC and procure the development, comprehensive validation, or adequate recalibration of available models.

### Implications for clinical practice

Several international societies and clinical guidelines have signaled the need to guide cardiovascular treatment and other prevention interventions based on absolute risk for which prognostic models are paramount 27, 49, 50, 51, 52, 53, 54. In particular, risk stratification based on these models contribute to allocation of health resources in a cost-effective manner, allowing individuals with higher global risk to receive adequate treatment and avoiding overtreatment in individuals at low risk 27, 49, 50, 51, 52, 53, 54.

In Chile, the tables of cardiovascular risk stratification adapted by Icaza et al. [31] were adopted by the Ministry of Health and have been used in the public primary health system since 2009 [54]. In 2013, Kunstmann et al. [55] conducted a validation of the Chilean tables, confirming that they are useful in the prediction of coronary and cerebrovascular events. Therefore, the Chilean tables continue to be used to estimate the global risk of cardiovascular diseases at individual level in Chile [55].

The fact that no cardiovascular prognostic models have been developed in LAC, and the fact that attempts to test the accuracy of available prognostic models in LAC have faced several limitations, puts LAC in a disadvantaged position to successfully lessen the burden of cardiovascular diseases. This review could not strongly recommend any available prognostic model to be used across LAC settings. Notwithstanding, acknowledging their limitations, weighing the pros and cons, and reaching an informed decision with the patient, country-specific models could be used after adequate recalibration.

## Conclusions

No cardiovascular prognostic model has been developed in or for the LAC region. The evaluation of available prognostic models signals to several limitations, and their prediction accuracy is questionable particularly regarding calibration, albeit discrimination was acceptable in most cases. Advancement of cardiovascular prognosis research might contribute to improve the allocation of scarce resources to people who need them the most, thus fostering the prevention of cardiovascular diseases in this world region and to achieve this goal, appropriate cardiovascular risk prediction is needed in the LAC region.
