# Supplementary material for: Cardiovascular Disease Prognostic Models in Latin America and the Caribbean: A Systematic Review
Source: Glob Heart. 2019 Mar;14(1):81–93. doi: 10.1016/j.gheart.2019.03.001 (PMC6499414; doi:10.1016/j.gheart.2019.03.001)
Supplement: Online Appendix [file mmc1.rtf]

CARDIOVASCULAR DISEASE PROGNOSTIC MODELS IN LATIN AMERICA AND THE CARIBBEAN: A SYSTEMATIC REVIEW


Supplementary Material


Contents
Training of reviewers before titles and abstract screening	2
Search Terms – LILACS & SCIELO	3
Search Terms – SCOPUS	4
Search Terms – OVID	5
PRISMA Checklist	8
Details for preparation of Figure 1 	11
Online Table 1: Discrimination estimates by risk prediction model as reported in the reviewed reports. 	12
PRISMA Flow Diagram 	13
Extraction form (following CHARMS framework) 	13
Risk of bias (PROBAST) 	14

CARDIOVASCULAR DISEASE PROGNOSTIC MODELS IN LATIN AMERICA AND THE CARIBBEAN: A SYSTEMATIC REVIEW


Training of reviewers before titles and abstract screening


On May 14^th^ 2018 a preliminary search using the same search terms was conducted. One hundred references were randomly selected and split into two groups of fifty references each. These groups served for training and standardization purposes between the reviewers who studied the titles and abstracts. The agreement between the reviewers was high.


Training 1 (Agreement: 0.94; Kappa: 0.93)


Reviewer 1 (RMCL)


Reviewer 2 (NP)
3
47
4	46	50


Training 2 (Agreement: 0.94; Kappa: 0.94)


Reviewer 1 (RMCL)


Reviewer 2 (NP)
3
47
0	50	50

CARDIOVASCULAR DISEASE PROGNOSTIC MODELS IN LATIN AMERICA AND THE CARIBBEAN: A SYSTEMATIC REVIEW


Search Terms – LILACS & SCIELO


Search terms (in Spanish) as they were included in these search engines.

((evaluación del riesgo coronario) OR (evaluación del riesgo cardiovasc$) OR (evaluación del riesgo de enfermedad cardiaca) OR (evaluación del riesgo de enfermedad coronaria) OR (evaluación del riesgo cardiovasc$) OR (evaluación del riesgo cv) OR (riesgo de enfermedad cardiaca) OR (score de riesgo cardiovasc$)) OR (((enfermedad coronaria) OR (enfermedad$ cardiovasc$) OR (enfermedad cardiaca) OR (infarto de miocardio) OR (riesgo cardiovasc$) OR (riesgo coronario$) OR (accidente cerebrovascular$) OR (isquemia cerebral$) OR (accidente cerebrovascular) OR (cerebrovascular) OR (circulación cerebrovascular)) AND (función de riesgo) OR (evaluación del riesgo) OR (funcion$ de riesgo) OR (ecuacion de riesgo) OR (tabla de riesgos$) OR (herramienta de ajuste de riesgo) OR (función de evaluación de riesgo) OR (asesor de riesgo) OR (cálculo de riesgo) OR (calculadora de riesgo) OR (motor de riesgo) OR (umbral de riesgo) OR (método de calificación de riesgo) OR (esquema de puntuación) OR (sistema de puntuación de riesgo) OR (predicción de riesgo) OR (algoritmo de riesgo) OR (modelo predictivo) OR (predicción de riesgo) OR (modelo de predicción) OR (instrumento predictivo) OR (proyecto$ riesgo)) OR ((tabla de Nueva Zeland$) OR (tabla de Sheffield) OR (guia de Dund$) OR (calificacion de BRHS) OR (BRHS riesgo) OR (riesgo de Copenhague) OR (framingham) OR (framingham riesgo) OR (ecuacion de framingham) OR (framingham modelo$) OR (Busselton risk$) OR (Busselton score$) OR (erica risk score$) OR (framingham scor$) OR (Dund$ scor$) OR (BRHS scor$) OR (British Regional Heart study scor$) OR (indice de riesgo BRHS) OR (indice de riesgo Dund$) OR (riesgo de framingham) OR (riesgo Copenhague)) AND ("Antigua and Barbuda" OR "Argentina" OR "Bahamas" OR "Barbados" OR "Belize" OR "Bolivia" OR "Brazil" OR "United States Virgin Islands" OR "British Virgin Islands" OR "Chile" OR "Colombia" OR "Costa Rica" OR "Cuba" OR "Dominica" OR "Dominican Republic" OR "EcuadOR" OR "El SalvadOR" OR "Grenada" OR "Guatemala" OR "Guyana" OR "Haiti" OR "Honduras" OR "Jamaica" OR "Mexico" OR "Nicaragua" OR "Panama" OR "Paraguay" OR "Peru" OR "Puerto Rico" OR "Saint Kitts and Nevis" OR "Saint Lucia" OR "Saint Vincent and the Grenadines" OR "Suriname" OR "Trinidad and Tobago" OR "West Indies" OR "Uruguay" OR "Venezuela" OR "Latin America" OR latin amer$ OR "South America" OR south amer$ OR "Central America" OR central amer$ OR "Caribbean Region")

CARDIOVASCULAR DISEASE PROGNOSTIC MODELS IN LATIN AMERICA AND THE CARIBBEAN: A SYSTEMATIC REVIEW


Search Terms – SCOPUS


Search terms as they were included in this search engine.

ALL(framingham scor*) OR ALL(dundee scor*) OR ALL(brhs scor*) OR ALL(British Regional Heart study risk scor*) OR ALL(brhs risk scor*) OR ALL(dundee risk scor*) OR ALL(framingham guideline*) OR ALL(framingham risk?) OR ALL(new zealand table*) OR ALL(ncep guideline?) OR ALL(smac guideline?) OR ALL(copenhagen risk?)) OR (ALL(cardiovascular diseases) OR ALL(coronary disease) OR ALL(cardiovascular disease*) OR ALL(heart disease*) OR ALL(coronary disease*) OR ALL(myocardial infarction*) OR ALL(cardiovascular risk?) OR ALL(coronary risk?) OR ALL(hypertension) OR ALL(hyperlipidemia)) AND (ALL(decision support techniques) OR ALL(Diagnosis, Computer- Assisted) OR ALL(Decision Support Systems,Clinical) OR ALL(algorithms) OR ALL(algorithm?) OR ALL(algorythm?) OR ALL(decision support?) OR ALL(predictive model?) OR ALL(treatment decision?) OR ALL(scoring method*) OR ALL((prediction* adj3 method*))) AND (ALL(Risk Factors) OR ALL(Risk Assessment) OR ALL((risk? adj1 assess*)) OR ALL(risk factor?)) OR (ALL(risk function) OR ALL(Risk Assessment) OR ALL(risk functions) OR ALL(risk equation*) OR ALL(risk chart?) OR ALL((risk adj3 tool*)) OR ALL(risk assessment function?) OR ALL(risk assessor) OR ALL(risk appraisal*) OR ALL(risk calculation*) OR ALL(risk calculator*) OR ALL(risk factor* calculator*) OR ALL(risk factor* calculation*) OR ALL(risk engine*) OR ALL(risk equation*) OR ALL(risk table*) OR ALL(risk threshold*) OR ALL(risk disc?) OR ALL(risk disk?) OR ALL(risk scoring method?) OR ALL(scoring scheme?) OR ALL(risk scoring system?) OR ALL(risk prediction?) OR ALL(risk algorith*) OR ALL(prediction model*) OR ALL(predictive instrument?) OR ALL(project* risk?) OR ALL(cdss)) AND (ALL(stroke) OR ALL(cerebrovascular accident*) OR ALL(brain schemia*) OR ALL(Stroke) OR ALL(cerebrovascular) OR ALL(Cerebrovascular Circulation)) OR (ALL(decision support techniques) OR ALL(Diagnosis, Computer-Assisted) OR ALL(Decision Support Systems,Clinical) OR ALL(algorithms) OR ALL(algorithm?) OR ALL(algorythm?) OR ALL(decision support?) OR ALL(predictive model?) OR ALL(treatment decision?) OR ALL(scoring method*) OR ALL((prediction* adj3 method*))) AND (ALL(Risk Factors) OR ALL(Risk Assessment) OR ALL((risk? adj1 assess*)) OR ALL(risk factor?)) AND (ALL(stroke) OR ALL(cerebrovascular accident*) OR ALL(brain ischemia*) OR ALL(Stroke) OR ALL(cerebrovascular) OR ALL(Cerebrovascular Circulation)) AND ("Antigua and Barbuda" OR "Argentina" OR "Bahamas" OR "Barbados" OR "Belize" OR "Bolivia" OR "Brazil" OR "United States Virgin Islands" OR "British Virgin Islands" OR "Chile" OR "Colombia" OR "Costa Rica" OR "Cuba" OR "Dominica" OR "Dominican Republic" OR "Ecuador" OR "El Salvador" OR "Grenada" OR "Guatemala" OR "Guyana" OR "Haiti" OR "Honduras" OR "Jamaica" OR "Mexico" OR "Nicaragua" OR "Panama" OR "Paraguay" OR "Peru" OR "Puerto Rico" OR "Saint Kitts and Nevis" OR "Saint Lucia" OR "Saint Vincent and the Grenadines" OR "Suriname" OR "Trinidad and Tobago" OR "West Indies" OR "Uruguay" OR "Venezuela" OR "Latin America" OR latin amer* OR "South America" OR south amer* OR "Central America" OR central amer* OR "Caribbean Region") AND NOT DBCOLL(medl)


Search Terms – OVID


Search terms as included in OVID, encompassing Embase and Medline.

01	chd risk assessment$.mp.	
02	cvd risk assessment$.mp.	
03	heart disease risk assessment$.mp.	
04	coronary disease risk assessment$.mp.	
05	cardiovascular disease risk assessment$.mp.	
06	cardiovascular risk assessment$.mp.	
07	cv risk assessment$.mp.	
08	cardiovascular disease$ risk assessment$.mp.	
09	coronary risk assessment$.mp.	
10	coronary risk scor$.mp.	
11	heart disease risk scor$.mp.	
12	chd risk scor$.mp.	
13	cardiovascular risk scor$.mp.	
14	cardiovascular disease$ risk scor$.mp.	
15	cvd risk scor$.mp.	
16	cv risk scor$.mp.	
17	1 or 2 or 3 or 4 or 5 or 6 or 7 or 8 or 9 or 10 or 11 or 12 or 13 or 14 or 15 or 16	
18	cardiovascular diseases/	
19	coronary disease/	
20	cardiovascular disease$.mp.	
21	heart disease$.mp.	
22	coronary disease$.mp.	
23	myocardial infarction$.mp.	
24	cardiovascular risk?.mp.	
25	coronary risk?.mp.	
26	exp hypertension/	
27	exp hyperlipidemia/	
28	18 or 19 or 20 or 21 or 22 or 23 or 24 or 25 or 26 or 27	
29	risk function.mp.	
30	Risk Assessment/mt	
31	risk functions.mp.	
32	risk equation$.mp.	
33	risk chart?.mp.	
34	(risk adj3 tool$).mp.	
35	risk assessment function?.mp.	
36	risk assessor.mp.	
37	risk appraisal$.mp.	
38	risk calculation$.mp.	
39	risk calculator$.mp.	
40	risk factor$ calculator$.mp.	
41	risk factor$ calculation$.mp.	
42	risk engine$.mp.	
43	risk equation$.mp.	
44	risk table$.mp.	


45	risk threshold$.mp.	
46	risk disc?.mp.	
47	risk disk?.mp.	
48	risk scoring method?.mp.	
49	scoring scheme?.mp.	
50	risk scoring system?.mp.	
51	risk prediction?.mp.	
52	risk algorith$.mp.	
53	prediction model$.mp.	
54	predictive instrument?.mp.	
55	project$ risk?.mp.	
56	cdss.mp.	
57	29 or 30 or 31 or 32 or 33 or 34 or 35 or 36 or 37 or 38 or 39 or 40 or 41 or 42 or 43
or 44 or 45 or 46 or 47 or 48 or 49 or 50 or 51 or 52 or 53 or 54 or 55 or 56	
58	28 and 57	
59	17 or 58	
60	new zealand chart$.mp.	
61	sheffield table$.mp.	
62	procam.mp.	
63	General Rule to Enable Atheroma Treatment.mp.	
64	dundee guideline$.mp.	
65	shaper scor$.mp.	
66	(brhs adj3 score$).mp.	
67	(brhs adj3 risk$).mp.	
68	copenhagen risk.mp.	
69	precard.mp.	
70	(framingham adj1 (function or functions)).mp.	
71	(framingham adj2 risk).mp.	
72	framingham equation.mp.	
73	framingham model$.mp.	
74	(busselton adj2 risk$).mp.	
75	(busselton adj2 score$).mp.	
76	erica risk score$.mp.	
77	framingham scor$.mp.	
78	dundee scor$.mp.	
79	brhs scor$.mp.	
80	British Regional Heart study risk scor$.mp.	
81	brhs risk scor$.mp.	
82	dundee risk scor$.mp.	
83	framingham guideline$.mp.	
84	framingham risk?.mp.	
85	new zealand table$.mp.	
86	ncep guideline?.mp.	
87	smac guideline?.mp.	
88	copenhagen risk?.mp.	
89	60 or 61 or 62 or 63 or 64 or 65 or 66 or 67 or 68 or 69 or 70 or 71 or 72 or 73 or 74
or 75 or 76 or 77 or 78 or 79 or 80 or 81 or 82 or 83 or 84 or 85 or 86 or 87 or 88	
90	59 or 89	


91	exp decision support techniques/	
92	Diagnosis, Computer-Assisted/	
93	Decision Support Systems,Clinical/	
94	algorithms/	
95	algorithm?.mp.	
96	algorythm?.mp.	
97	decision support?.mp.	
98	predictive model?.mp.	
99	treatment decision?.mp.	
100	scoring method$.mp.	
101	(prediction$ adj3 method$).mp.	
102	91 or 92 or 93 or 94 or 95 or 96 or 97 or 98 or 99 or 100 or 101	
103	Risk Factors/	
104	exp Risk Assessment/	
105	(risk? adj1 assess$).mp.	
106	risk factor?.mp.	
107	103 or 104 or 105 or 106	
108	28 and 102 and 107	
109	90 or 108	
110	stroke.mp.	
111	cerebrovascular accident$.mp.	
112	brain ischemia$.mp.	
113	exp Stroke/	
114	cerebrovascular.mp. or exp Cerebrovascular Circulation/	
115	110 or 111 or 112 or 113 or 114	
116	57 and 115	
117	102 and 107 and 115	
118	116 or 117	
119	109 or 118	
120	("Antigua and Barbuda" or "Argentina" or "Bahamas" or "Barbados" or "Belize" or "Bolivia" or "Brazil" or "United States Virgin Islands" or "British Virgin Islands" or
"Chile" or "Colombia" or "Costa Rica" or "Cuba" or "Dominica" or "Dominican Republic" or "Ecuador" or "El Salvador" or "Grenada" or "Guatemala" or "Guyana" or "Haiti"  or  "Honduras"  or  "Jamaica"  or  "Mexico"  or  "Nicaragua"  or  "Panama"  or
"Paraguay" or "Peru" or "Puerto Rico" or "Saint Kitts and Nevis" or "Saint Lucia" or "Saint Vincent and the Grenadines" or "Suriname" or "Trinidad and Tobago" or
"West Indies" or "Uruguay" or "Venezuela" or "Latin America" or latin amer$ or "South America" or south amer$ or "Central America" or
central amer$ or "Caribbean Region").mp.	
121	119 and 120	
122	remove duplicates from 121	


PRISMA Checklist


Section/topic	
#	
Checklist item	Reported on page
#	
TITLE		
Title	1	Identify the report as a systematic review, meta-analysis, or both.	
01	
ABSTRACT		
Structured summary	2	Provide a structured summary including, as applicable: background; objectives; data sources; study eligibility criteria, participants, and interventions; study appraisal and synthesis methods; results; limitations; conclusions and implications of key findings; systematic review registration number.	


02	
INTRODUCTION		
Rationale	3	Describe the rationale for the review in the context of what is already known.	
04	
Objectives	4	Provide an explicit statement of questions being addressed with reference to participants, interventions, comparisons, outcomes, and study design (PICOS).	
04	
METHODS		
Protocol and registration	5	Indicate if a review protocol exists, if and where it can be accessed (e.g., Web address), and, if available, provide registration information including registration number.	
05	
Eligibility criteria	6	Specify study characteristics (e.g., PICOS, length of follow- up) and report characteristics (e.g., years considered, language, publication status) used as criteria for eligibility, giving rationale.	

05	
Information sources	7	Describe all information sources (e.g., databases with dates of coverage, contact with study authors to identify additional studies) in the search and date last searched.	
05	
Search	8	Present full electronic search strategy for at least one database, including any limits used, such that it could be repeated.	
05-06	
Study selection	9	State the process for selecting studies (i.e., screening, eligibility, included in systematic review, and, if applicable, included in the meta-analysis).	
05-06	
Data collection process	10	Describe method of data extraction from reports (e.g., piloted forms, independently, in duplicate) and any processes for obtaining and confirming data from investigators.	

05-06	


Data items	11	List and define all variables for which data were sought (e.g., PICOS, funding sources) and any assumptions and simplifications made.	
05-06	
Risk of bias in individual studies	12	Describe methods used for assessing risk of bias of individual studies (including specification of whether this was done at the study or outcome level), and how this information is to be used in any data synthesis.	

06	
Summary measures	13	State the principal summary measures (e.g., risk ratio, difference in means).	
06	
Synthesis of results	14	Describe the methods of handling data and combining results of studies, if done, including measures of consistency (e.g., I^2^) for each meta-analysis.	
NA	
Risk of bias across studies	15	Specify any assessment of risk of bias that may affect the cumulative evidence (e.g., publication bias, selective reporting within studies).	
NA	
Additional analyses	16	Describe methods of additional analyses (e.g., sensitivity or subgroup analyses, meta-regression), if done, indicating which were pre-specified.	
NA	
RESULTS		
Study selection	17	Give numbers of studies screened, assessed for eligibility, and included in the review, with reasons for exclusions at each stage, ideally with a flow diagram.	

07	
Study characteristics	18	For each study, present characteristics for which data were extracted (e.g., study size, PICOS, follow-up period) and provide the citations.	
07	
Risk of bias within studies	19	Present data on risk of bias of each study and, if available, any outcome level assessment (see item 12).	
08	
Results of individual studies	20	For all outcomes considered (benefits or harms), present, for each study: (a) simple summary data for each intervention group (b) effect estimates and confidence intervals, ideally with a forest plot.	

08	
Synthesis of results	21	Present results of each meta-analysis done, including confidence intervals and measures of consistency.	
08	
Risk of bias across studies	22	Present results of any assessment of risk of bias across studies (see Item 15).	
NA	
Additional analysis	23	Give results of additional analyses, if done (e.g., sensitivity or subgroup analyses, meta-regression [see Item 16]).	
NA	
DISCUSSION		
Summary of evidence	24	Summarize the main findings including the strength of evidence for each main outcome; consider their relevance to key groups (e.g., healthcare providers, users, and policy makers).	

09	
Limitations	25	Discuss limitations at study and outcome level (e.g., risk of bias), and at review-level (e.g., incomplete retrieval of	
09-10	


		identified research, reporting bias).		
Conclusions	26	Provide a general interpretation of the results in the context of other evidence, and implications for future research.	
12	
FUNDING		
Funding	27	Describe sources of funding for the systematic review and other support (e.g., supply of data); role of funders for the systematic review.	

01	

From: Moher D, Liberati A, Tetzlaff J, Altman DG, The PRISMA Group (2009). Preferred Reporting Items for Systematic Reviews and Meta-Analyses: The PRISMA Statement. PLoS Med 6(7): e1000097. doi:10.1371/journal.pmed1000097
For more information, visit: www.prisma-statement.org.


Details for preparation of Figure 1
The map was downloaded from http://yourfreetemplates.com: Creative Commons' license that is Attribution-NoDerivatives 4.0 International (CC BY-ND 4.0). And every template you download from yourfreetemplates.com is the intellectual property of and is owned by us. Free to make any necessary modification(s) to our PowerPoint templates to fit your purposes, personally, educationally, or even commercially. Not allowed to i) Upload any of our PowerPoint templates, modified or unmodified, on a diskette, CD, your website or content share ones like Slideshare , Scribd, YouTube, LinkedIn, and Google+ etc; ii) Offer them for redistribution or resale in any form without prior written consent from yourfreetemplates.com.

CARDIOVASCULAR DISEASE PROGNOSTIC MODELS IN LATIN AMERICA AND THE CARIBBEAN: A SYSTEMATIC REVIEW


Online Table 1: Discrimination estimates by risk prediction model as reported in the reviewed reports.

	Polenz, 2015	Munoz, 2014	Gulayin, 2018	Sandoya, 2009	Acevedo, 2017	Jimenez-Corona, 2009	Joseph, 2018	D'Angostino, 2001	
CHA2DS2VASc	0.62 (0.58-0.67)								
Framingham		0.66 (0.63-0.69)	0.72		0.60 (0.52-0.74)	Not reported.		0.72	
Men				0.76 (0.69-0.82)					
Women				0.67 (0.56-0.78)					
PROCAM		0.58 (0.52-0.64)							
PROCAM (sex)		0.75 (0.71-0.77)							
CUORE			0.75						
Globorisk			0.75						
ACC/AHA			0.74		0.78 (0.68-0.84)				
NL-IHRS							0.72 (0.69-0.75)		
FC-IHRS							0.74 (0.71-0.77)		


12


PRISMA Flow Diagram


Records identified through database searching = 2506


Ovid (EMBASE & Medline) = 522 (520) Scopus = 1740 (1723)
SciElo = 101 (49)
Additional records identified through other sources
(n = 0)


Records after duplicates removed (n = 2420)


Records screened (n = 2420)
Records excluded (n = 2403)


Full-text articles assessed for eligibility
(n = 17)


Studies included in qualitative synthesis (n = 08)


Studies included in quantitative synthesis (meta-analysis)
(n = 0)
Full-text articles excluded, with reasons
(n = 09)

Not prospective design = 04

No assessment of the prediction model but comparison between men and women = 01

All-cause mortality as outcome = 01 Duplicate = 01
Not Latin-American populations = 02


13

CARDIOVASCULAR DISEASE PROGNOSTIC MODELS IN LATIN AMERICA AND THE CARIBBEAN: A SYSTEMATIC REVIEW


Risk of bias (PROBAST)


Y=Yes; PY=Probably yes; N=No; PN=Probably no; NI=No information; n/a=not applicable (only apply for studies developing new models).


14

Applicability (PROBAST)

Study	Participants	Predictors	Outcome	
Polenz, 2015	High	Low	Low	
Munoz, 2014	High	Low	Low	
Gulayin, 2018	Low	Low	Low	
Sandoya, 2009	High	Low	Low	
Acevedo, 2017	High	Low	Low	
Jiménez-Corona, 2009	Low	Low	Low	
Joseph, 2018	Low	Low	Low	
D'Agostino, 2001	Low	Low	Low	

Low = Low concern for applicability; High = High concern for applicability; Unclear = Unclear concern for applicability.

Extraction form (following CHARMS framework)
